# Trade-offs between *Xylella fastidiosa* vector control and conservation of beneficial arthropods in Mediterranean olive groves

**DOI:** 10.1093/jee/toag023

**Published:** 2026-02-25

**Authors:** Ilaria Laterza, Gianvito Ragone, Biagio Tedone, Nicola Bodino, Enrico de Lillo, Daniele Cornara, Giovanni Tamburini

**Affiliations:** Department of Soil, Plant and Food Sciences (DiSSPA—Entomology and Zoology), University of Bari, Bari, Italy; Department of Soil, Plant and Food Sciences (DiSSPA—Entomology and Zoology), University of Bari, Bari, Italy; Regional Plant Protection Service-Apulia Region, Lungomare Nazario Sauro, Bari, Italy; Department of Soil, Plant and Food Sciences (DiSSPA—Entomology and Zoology), University of Bari, Bari, Italy; Department of Agricultural, Forest and Food Sciences (DISAFA), University of Turin, Turin, Italy; Department of Soil, Plant and Food Sciences (DiSSPA—Entomology and Zoology), University of Bari, Bari, Italy; Department of Soil, Plant and Food Sciences (DiSSPA—Entomology and Zoology), University of Bari, Bari, Italy; Department of Soil, Plant and Food Sciences (DiSSPA—Entomology and Zoology), University of Bari, Bari, Italy

**Keywords:** agricultural intensification, carabid beetles, egg predation, pollinators, spiders

## Abstract

*Xylella fastidiosa* is a xylem-limited gram-negative bacterium responsible for significant crop yield losses worldwide. The sequence type ST53 is recognized as the causal agent of the olive quick decline syndrome, currently threatening olive production in southern Europe, where the olive-to-olive transmission of the bacterium is primarily mediated by a xylem-sap feeder: the meadow spittlebugs *Philaenus spumarius*. Mandatory control measures, such as the mechanical removal of ground cover, are currently enforced to reduce juvenile vector populations and curb the spread of the pathogen. However, the long-term sustainability of this control strategy in olive groves remains uncertain, particularly regarding its impact on beneficial arthropods. Here, we investigated how seasonal variation in ground cover within olive groves influences abundance of adult *X. fastidiosa* vectors, beneficial arthropods (i.e., wild pollinators, spiders, and carabids) and predation of *P. spumarius* eggs in Mediterranean olive groves, while also accounting for surrounding agricultural matrix. Our results showed that the importance of herbaceous vegetation conditions varies across seasons with early spring and spring ground cover emerging as the strongest predictors for most functional groups, while spider populations responded more strongly to summer conditions. Overall, ground cover promoted adult *X. fastidiosa* vectors, wild pollinators, spiders and carabids depending on the season, whereas complex landscapes enhanced both pollinator activity and egg predation. Intensive ground cover management appears to generate trade-offs between vector control and the conservation of beneficial organisms in Mediterranean agroecosystems, but adjusting the timing of vegetation removal or adopting alternative ground cover management may help to mitigate these conflicts.

## Introduction

Mediterranean olive groves have been facing a major phytosanitary challenge over the last decades: the spread of *Xylella ­fastidiosa* (Wells et al. 1987), a xylem gram-negative bacterium recognized as the causal agent (sequence type ST53) of the Olive Quick Decline Syndrome (OQDS) (Saponari et al. 2013, Saponari et al. 2017). The bacterium is also responsible for significant yield losses in many economically important crops worldwide (Sicard et al. 2018). The first occurrence of *X. ­fastidiosa* subsp. *pauca* (sequence type ST53) in Europe was recorded in Apulia region, southern Italy, in 2013, where it has severely impacted olive production and agricultural heritage in the region (Schneider et al. 2020). The primary vector ­mediating olive-to-olive transmission of the bacterium in Europe is the highly polyphagous meadow spittlebug *Philaenus spumarius* L. (Hemiptera: Aphrophoridae), which is the most abundant xylem sap-feeding hemipteran in the area (Cornara et al. 2017). Other species competent for *X. fastidiosa* transmission ­common in olive orchards in the Apulia Region are the spittlebugs ­*Neophilaenus campestris* (Fallén) and *Philaenus italosignus* (Drosopoulos and Remane) (Hemiptera: Aphrophoridae) ­(Cavalieri et al. 2019). The three spittlebug species share similar seasonal phenology in southern Italy: the juvenile stages are characterized by limited mobility (i.e., few meters) and appear in olive groves from March to May, colonizing exclusively herbaceous plants. Adults are quite mobile (i.e., able to fly ≈ 500 m in 30 min) (Lago et al. 2021, Casarin et al. 2023), and migrate to olive canopies as temperatures rise and ground cover dries, usually between May and October (Cornara et al. 2017, Bodino et al. 2019). Later in the season (i.e., autumn), they return on the dry grasses to lay eggs, which are the overwintering stage of these insects (Cornara et al. 2018, Bodino et al. 2023). These vectors undoubtedly play a key role in the spread of the bacterium in southern Europe, and their control via agronomic or chemical practices represents a key component of current strategies to reduce *X. fastidiosa* spread. At the same time, the contribution of biological regulation by natural antagonists to control vector population under field conditions remains largely unresolved, with only limited information available on predation of adults and nymphs by generalist predators (e.g., carabid beetles, birds) (Harper and Whittaker 1976), even less is known about natural enemies of the egg stage, with the exception of recently documented parasitoids (Mesmin et al. 2020). Vector management in olive groves through both mechanical and chemical means is currently mandatory in *X. fastidiosa* outbreak areas (Apulian Phytosanitary Service 2024). These control strategies include the removal of ground cover in early spring, targeting vector juveniles, and the application of broad-spectrum insecticides in early summer to control adults before canopy colonization (Apulian Phytosanitary Service 2024). Yet, the long-term sustainability of intensive ground cover removal remains uncertain (Labadessa et al. 2024), as the disturbance caused by tillage and frequent mowing can negatively impact farmland biodiversity and reduce the provision of key ecosystem services across cropping systems (e.g., Winter et al. 2018, Lee et al. 2019, Rowen et al. 2020). Research conducted in olive groves reports that an intensive ground cover management negatively affects pollinator communities (Martínez-Núñez et al. 2019, Cano et al. 2024), spider abundance and canopy arthropods (Paredes et al. 2013, Vasconcelos et al. 2022), but has limited effects on herbivore pests (Paredes et al. 2015). In this context, understanding the effects of ground cover management on both vector control and multiple beneficial arthropod groups simultaneously, is crucial to plan efficient and sustainable control strategies within olive groves.

The composition of agricultural landscapes also appears to play an important role in the spatial dynamics of *X. fastidiosa* vectors (Santoiemma et al. 2019) and potentially on pathogen spread (Bajocco et al. 2023). The presence of olive groves, grasslands and forests in the landscape has been associated with higher abundance of *P. spumarius* (Santoiemma et al. 2019, Cappellari et al. 2022, Gallego et al. 2023), probably because these habitats provide suitable host plants, and are characterized by lower disturbance compared to others (e.g., vineyards, urban gardens), hence favoring spittlebug populations. Seminatural habitats also support several beneficial organisms, and complex landscapes are generally expected to host more abundant and diverse communities of pollinators and natural enemies (Dainese et al. 2019, Bottero et al. 2023). It is still not clear whether abundant seminatural habitats in agricultural landscapes would support both beneficial arthropod and vector populations, potentially generating a trade-off between biodiversity conservation and *X. fastidiosa* vector control.

Here, we investigated the impacts of seasonal ground cover management on abundance of adult *X. fastidiosa* vectors, wild pollinators, spiders, carabid beetles and on *P. spumarius* egg predation in 28 olive groves selected along a gradient in landscape simplification across the Apulia region, the largest olive-growing region in Italy (Orlandi et al. 2012). Although ground cover management is primarily expected to influence juvenile stages, we focused on adult vectors as they represent the net outcome of these control measures and are directly responsible for pathogen transmission. In addition to vectors, we assessed multiple groups of beneficial arthropods that contribute to key ecosystem services, such as biological control and pollination, even if these services are not directly linked to olive production or *X. fastidiosa* epidemiology. Olive groves cover extensive areas in Mediterranean landscapes and, as perennial and structurally stable agroecosystems, can function as important habitats for beneficial organisms. Consequently, their management can have far-reaching effects on farmland biodiversity and on ecosystem services provided both within olive groves (e.g., biological pest control) and in adjacent cropping systems (e.g., pollination for insect-pollinated crops). In this context, spiders and carabid beetles represent dominant groups of generalist predators in agricultural soils and can contribute to the regulation of herbivorous insects, including *X. fastidiosa* vectors, through predation on juvenile stages or eggs. We hence examined how ground cover removal across seasons (i.e., early spring, spring, summer, autumn) and years (i.e., 2022, 2023) influences multiple organisms to potentially identify optimal timings for soil operations that suppress vectors while limiting harm to beneficial arthropods. We hypothesized that vectors and beneficial arthropods would exhibit similar responses to soil disturbance and landscape composition. Specifically, we predicted that (i) ground cover removal would reduce vector populations in olive groves while also negatively affecting beneficial arthropods and egg predation; (ii) the timing of ground cover removal would mediate these responses, with soil operations conducted in different seasons exerting contrasting effects on vectors and beneficial arthropod groups; and (iii) landscapes rich in seminatural habitats would support more abundant populations of both vectors and beneficial arthropods.

## Materials and Methods

The study combined arthropod field surveys with ground vegetation data derived from an independent regional monitoring program (Fig. S1). Arthropod sampling was conducted in spring 2023, while the egg predation experiment took place between December 2023 and January 2024. Ground vegetation cover data were obtained from a biweekly monitoring program coordinated by the Apulian Phytosanitary Service and were used to characterize seasonal ground cover conditions preceding sampling.

### Ground Cover Vegetation and Landscape Characterization

The survey was conducted between April 2023 and January 2024 across the agricultural landscapes of Bari, Taranto, and Barletta-Andria-Trani districts in the Apulia region (southern Italy) (Fig. S1). The study area is characterized by a Mediterranean semiarid climate (mean annual precipitation = 675 mm, mean annual temperature = 17.6 °C) and a high cover of perennial crops (mainly olive groves, but also fruit orchards and vineyards; c. 64%), interspersed with seminatural areas (i.e., mainly grasslands; c. 8%).

We selected a total of 28 olive groves along a gradient in the cover of seminatural habitats within the surrounding landscape (1 km radius, range = 0% to 78%), all characterized by broadly comparable overall productive and management system and located within a relatively homogeneous agro-climatic context. Landscape composition around each olive grove was quantified in QGIS at 3 spatial scales (i.e., 250, 500, 1000 m radii) measuring the cover of seminatural habitats (i.e., grasslands and small forest patches) and of olive groves. These variables were chosen because they represent the dominant land uses in the study area and are closely linked to the ecology of the studied organisms. Seminatural habitats provide key resources for both vectors and beneficial arthropods (Cornara et al. 2021, Cappellari et al. 2022, Ragone et al. 2025), whereas olive groves have been shown to support vector populations (Santoiemma et al. 2019).

Ground vegetation cover data were collected in 2022 and 2023 as part of a large-scale regional monitoring campaign of *X. fastidiosa* vectors conducted by phytosanitary authorities (Regional Vector Monitoring Plan 2022). Olive groves were visited biweekly and ground cover was assessed at each visit in four 1 × 1 m randomly selected plots per grove, using four cover classes (0% to 24%, 25% to 49%, 50% to 74%, 75% to 100%). To evaluate the potential effects of ground cover removal performed at different times of the year, we calculated the average ground cover for each site across defined seasonal intervals in the year preceding the sampling (Fig. S1). We considered seven intervals in total, which were grouped into categories according to their approximate correspondence with calendar seasons: spring, summer and autumn of 2022 (21 April to 23 June, 24 June to 21 September, 22 September to 28 October, respectively), and early spring, spring, summer and autumn of 2023 (25 March to 23 May, 21 April to 23 June, 24 June to 21 September, 22 September to 28 October, respectively). Early spring 2023 was specifically included because it corresponds to the period of spittlebug juvenile development on herbaceous vegetation in the study area (Bodino et al. 2019).

### Vector and Beneficial Arthropod Sampling

The sampling of adult *X. fastidiosa* vectors and beneficial arthropods was conducted in May and June 2023. As mentioned before, we focused on adults as they are directly involved in pathogen transmission and they represent the net outcome of control measure focusing on ground cover removal. Adult vector abundance was assessed for each olive grove following standard protocols (EFSA et al. 2019). Specifically, the abundance of adult vectors (*P. spumarius, P. italosignus* and *N. campestris*) was recorded approximately every two weeks (average number of visits per site: 4.5 ± 0.11) by monitoring both ground cover and olive canopies. Vectors on ground cover were sampled using a sweep net (38 cm diameter), with four consecutive sweeps performed at 18 points (1 × 1 m) equally distributed in each site (i.e., 72 sweeps per site per visit on the ground). Vectors on canopies were sampled from 18 olive trees randomly selected in each site using a sweep net at four different point (one per cardinal point) per each olive tree (i.e., 72 sweeps per site per visit on the canopy). All specimens were identified in the field according to the EPPO Diagnostic Protocol PM 7/141 (Germain et al. 2020). For analysis, vector abundance was summed per sampling round at the site level and subsequently averaged across sampling rounds.

Pollinator surveys were conducted twice at each site, with two rounds spaced three weeks apart, once in the morning and once in the afternoon, between 8:00 AM and 4:00 PM. Pollinator abundance was recorded walking slowly for ∼10 min along two 100 m transects randomly selected in each site, counting all wild pollinators observed (i.e., butterflies, bumblebees, solitary bees, and hoverflies) (Potts et al. 2003). During each visit, flower cover (0 to 5 cover classes: 0% to 24%, 25% to 49%, 50% to 74%, 75% to 100%) was also estimated for each transect. Surveys were carried out under favorable climatic conditions, that is, warm and sunny days, with no strong winds (below Beaufort scale 5) and air temperature above 17 °C (McCravy 2018). For analysis, we calculated the total abundance of wild pollinators for each site.

Ground dwelling predators (i.e., spiders and carabid beetles) were monitored installing four pitfall traps per site (112 traps in total) placed at 25-m intervals along a central row of the olive grove. Pitfall traps were installed on 15-May-2023 and retrieved on 25-June-2023. Specifically, each pitfall trap consisted of one plastic cup (dimensions: 400 ml, 8 cm in diameter and 13 cm deep) buried flush to the soil surface and covered by a plastic dinner plate suspended 3 cm above each trap to prevent rainwater entry (Gallé et al. 2020, Geppert et al. 2024). Approximately 200 ml of 70% ethylene glycol was added to each trap for sample preservation. Arthropods were collected every 20 days (two rounds), stored in 70% ethanol and transported to the laboratory for visual inspection to assess the abundance of carabids and spiders. Since some traps were damaged by soil management operations (n = 33), ground dwelling predator abundance was calculated for each site as the mean number of spiders or carabid beetles collected per trap per day.

### Predation of *Philaenus spumarius′* Eggs

We measured the impact of studied factors on the potential biological control of *P. spumarius* eggs by natural enemies. A subset of olive groves (n = 15) was selected among those covering an adequate gradient of cover of seminatural habitats in the landscape in a range from 0% to 28%. We measured the predation of *P. spumarius* eggs as a proxy for natural biological control. Egg masses were obtained from *P. spumarius* adults collected in the field between September and October 2023, caged in plastic and mesh cages on 3-week-old vetch (*Vicia sativa* L.) and fava bean (*V. faba* L.), and reared in a screenhouse without temperature, photoperiod or humidity control. Dry pine needles were placed on the base of the cages and on the pots’ surface to facilitate oviposition. Cages were checked once per week from October to December 2023 to collect the egg masses on the dry pine needles. Prior to the exposure in the field, egg masses were checked under a dissecting microscope to verify their integrity and to check the presence of eggs inside. We selected intact egg masses and measured their length as a proxy for egg abundance. This methodology avoided the removal of the protective solid foam to count the eggs, a process that can alter egg viability (Weaver and King 1954). The strict correlation between egg mass length and number of eggs was confirmed on few examined egg masses (n = 22, *R*^2^ = 0.97). Finally, in December 2023, a total of 150 egg masses (10 per site) laid on individual dry pine needles were enclosed in rigid plastic nets (1 × 1 cm mesh) and placed along the edge of each site (Fig. S2), with ∼10 m between each egg mass. After one month, eggs masses were retrieved from the field, examined for predation damage, and re-measured in length to quantify the proportion of egg mass lost to predation (Fig. S2). To assess potential presence of parasitoids, the dry pine needles were placed individually on filter papers in Petri dishes at room temperature (20 ± 2 °C), with natural light (10:14, L:D). Filter papers were kept moist by adding drops of water when necessary (Mesmin et al. 2020). Finally, since no parasitoids were observed to emerge after four weeks, all eggs were dissected to verify the presence of parasitoid larvae or pupae that failed to develop and emerge. Egg predation data were averaged at the site level.

### Statistical Analysis

We analyzed the effects of ground cover and landscape composition on the abundance of *X. fastidiosa* vectors, abundance of beneficial arthropods and *P. spumarius* egg predation. Because of the high number of potentially correlated predictors at both the local scale (seven ground cover intervals across seasons and years) and the landscape scale (cover of olive groves and seminatural habitats at three spatial scales: six landscape variables) (Table S1), we adopted a two-step approach for predictor selection. First, for each response variable, we compared ground cover intervals from the 12-month period preceding sampling, as these were considered the most relevant. We tested summer 2022, autumn 2022, early spring 2023 and spring 2023 for vector, wild pollinator, spider and carabid abundance, and early spring 2023, spring 2023, summer 2023, and autumn 2023, for egg predation (Fig. S1). Each interval was tested in separate linear models including only that ground cover variable as a fixed effect, and the interval yielding the highest pseudo-*R*^2^ was retained for further analyses. Second, for each response variable we ran six additional models that combined the selected ground cover variable with one landscape predictor at a time. The model displaying the lowest AIC was considered as the best fitting model (Akaike 2011, Table S2). This data-driven approach avoided the a priori selection of specific temporal windows and focused on the year preceding biological sampling, which was both constrained by data availability and considered ecologically appropriate given the life cycles of the studied organisms.

The final linear models therefore included the best ground cover predictor, the best landscape predictor, and their interaction terms, with non-significant interactions removed to reduce complexity. For each model, the most appropriate family was chosen to meet model assumptions (i.e., Tweedie with a log link, nbinom1, and beta family). For wild pollinators, the average flower cover along transects was included as an offset because higher flower abundance could artificially inflate pollinator counts. As an offset, flower cover was treated as a fixed scaling term and was therefore not estimated as a model coefficient. All predictors were modeled as continuous covariates (1 df). Model assumptions were evaluated using simulated residuals in the DHARMa package, and variance inflation factors confirmed low collinearity among predictors, with all values below 1.02. We performed the analyses using the “glmmTMB” package (Magnusson et al. 2017) implemented in R version 2025.05.1 + 513. Full model details are reported in Table S3.

## Results

In total, we collected 757 *X. fastidiosa* adult vectors (539 *P. spumarius*, 218 *N. campestris* and 0 *P. italosignus*), 682 spiders, 251 carabid beetles and counted 203 wild pollinators. The average egg predation was 31.6%, and no parasitoids emerged from the exposed egg masses.

**Fig. 1.**
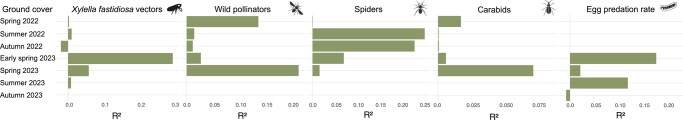
Results of model selection for choosing the ground cover as predictor for the different models. Models were run including cover ground vegetation measured at different seasonal periods (i.e., early spring, spring, summer and autumn) across years (2022, 2023), and compared using the pseudo-*R*^2^. Icons: Flaticon.com

The effects of ground vegetation cover varied across the time intervals considered, with the most influential periods differing among taxa. Early spring and spring 2023 were most often identified as the best predictors, explaining the highest variability in vector abundance, egg predation, and in the abundance of wild pollinators and carabid beetles, respectively. In contrast, spider abundance was best explained by ground cover from summer 2022 (Fig. 1).

**Fig. 2.**
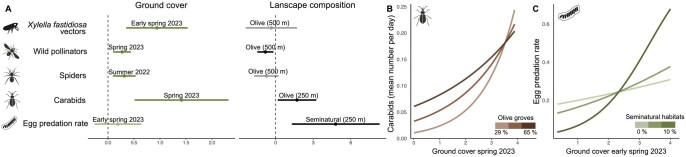
A) Effect of ground cover and landscape composition (model effect sizes and 95% CIs) on the abundance of *Xylella fastidiosa* vectors, wild pollinators, spiders (mean number of spiders per day), carabids (mean number of carabids per day) and *Philaenus spumarius* egg predation (proportion of missing eggs). Bold lines represent significant effect size (*P* < 0.05). B) Interactive effect of ground cover (spring 2023) and landscape composition (cover of olive groves, 250 m scale) on carabids. C) Interactive effect of ground cover (early spring 2023) and landscape composition (cover of seminatural habitats, 250 m scale) on egg predation. Icons: Flaticon.com

Overall, ground cover had a positive effect on studied variables (Table 1, Fig. 2). In particular, we found a direct positive effect of ground cover on the abundance of *X. fastidiosa* vectors, wild pollinators and spiders (Fig. 2A).

**Table 1.** Results of the best fitting linear models (lowest AIC) testing the effects of ground cover over the season (early spring 2023, spring 2023, summer 2022), landscape composition (cover of seminatural habitats or olive groves) and their interaction on *X. fastidiosa* vector abundance, beneficial arthropods (wild pollinators, spiders and carabids) and *P. spumarius* egg predation

Variable	Chisq	*P*-value
*Xylella fastidiosa* vectors		
**Ground cover (early spring 2023)**	**9.74**	**0.002**
% olive groves (500 m)	0.10	0.750
Wild pollinators		
**Ground cover (spring 2023)**	**9.72**	**0.002**
**% olive groves (500 m)**	**6.10**	**0.013**
Spiders		
**Ground cover (summer 2022)**	**7.85**	**0.005**
% olive groves (500 m)	2.15	0.143
Carabids		
**Ground cover (spring 2023)**	**4.07**	**0.044**
% olive groves (250 m)	1.09	0.297
**Ground cover (spring 2023) × % olive groves (250 m)**	**6.06**	**0.014**
Egg predation rate		
Ground cover (early spring 2023)	2.82	0.093
**% seminatural habitats (250 m)**	**7.98**	**0.005**
**Ground cover (early spring 2023) × % seminatural habitats (250 m)**	**4.23**	**0.040**

Significant variables and values are represented in bold.

Carabid beetle abundance and egg predation also increased with increasing ground cover, but the strength of these relationships depended on landscape composition at the 250 m scale (ground cover × landscape composition interaction). Specifically, the positive effect of ground cover on carabid abundance was strongest in complex landscapes (Fig. 2B), although abundant olive groves in the landscapes compensated for low ground cover. Egg predation responded most strongly to ground cover in landscapes with a higher proportion of seminatural habitats, whereas the relationship was weaker in more simplified landscapes (Fig. 2C).

Finally, landscape composition directly affected only wild pollinator abundance, showing a negative relationship with olive grove cover at the 500 m scale, whereas no significant landscape effects were observed for vectors or spiders.

## Discussion

This study investigated how seasonal variation in ground vegetation cover within olive groves influences abundance of *X. fastidiosa* vectors, beneficial arthropods and predation of *P. spumarius* eggs in Mediterranean olive groves, while also accounting for surrounding agricultural matrix. Overall, intensive ground cover management appears to generate trade-offs between vector control and the conservation of beneficial organisms (i.e., wild pollinators, spiders, and carabids) in ­Mediterranean agroecosystems, but adjusting the timing of vegetation removal may help to mitigate these conflicts. In this context, we found ground cover to generally promote *X. fastidiosa* vectors, wild pollinators, spiders, carabid beetles and egg predation, depending on the season and on the landscape context.

The removal of ground cover in spring has become mandatory in parts of Apulia region to reduce resident vector populations in olive groves in the attempt to limit the spread of *X. fastidiosa* (Cornara et al. 2018, Apulian Phytosanitary Service 2024). As expected, and in line with previous findings (e.g., Sanna et al. 2021), we observed higher ground cover to promote adult vector abundance, as herbaceous vegetation is essential to complete their life cycle (Weaver and King 1954). Early spring 2023 emerged as the best predictive period, likely because it coincides with the presence of nymphs on herbaceous hosts, which directly determines the size of adult populations observed in olive groves in the next June. Considering the nymphs’ limited dispersal capacity and their strong dependence on ground cover, removing ground vegetation cover during this window effectively reduce population abundance. By contrast, ground cover from the preceding seasons (i.e., spring, summer, autumn 2022, and spring 2023) showed considerably weaker predictive power for current vector abundance, suggesting an overriding importance of direct mechanical disturbance on nymphs, compared to any delayed, carry-over effects of ground cover conditions, for example during oviposition period in autumn. Importantly, our approach was designed to reflect the ground cover management under real-world farming conditions, where the timing and intensity of vegetation removal vary among farmers across the study area. As a consequence, the observed variation in ground cover represents natural management heterogeneity rather than a controlled experimental treatment, and some confounding effects related to site-specific characteristics cannot be fully excluded. Accordingly, our results should be interpreted as correlative rather than strictly causal. Moreover, because our study focused on adults collected only in spring 2023 (April to June), it remains uncertain whether spring vegetation management effectively limit overall vector population, including summer and autumn adults, and thus lowers infection risk. The influence of plant assemblages within olive groves on vector populations also remains unclear. Further research is needed to assess whether early-season interventions limit adult abundance on olive trees after spring, the critical period for pathogen spread. Finally, contrary to previous studies (Santoiemma et al. 2019, Cappellari et al. 2022), we found no response of *X. fastidiosa* vectors to landscape composition. This unexpected result might indicate different population dynamics of the pest in the study area, claiming for more comprehensive surveys in multiple sites and years.

As expected, ground cover had a positive effect on wild pollinator populations, likely because more abundant ground vegetation provides a richer supply of floral resources within olive groves (Martínez-Núñez et al. 2019). Although olive is a wind-pollinated crop, the structural complexity and stability of this agroecosystem offer strong potential for biodiversity conservation, with pollinator communities able to rely on resources within olive groves such as wild plants and nesting materials (Martínez‐Núñez et al. 2020, Rey et al. 2019). In this context, our results suggest that ground cover within olive groves may substantially support wild pollinators, potentially influencing the ecosystem services they provide across extensive areas, considering that pollinator abundance may reflect broad patterns in wild bee species richness (Potts et al. 2006, Marini et al. 2025). In our study, spring 2023 emerged as the most predictive period, coinciding with the peak of floral availability in Mediterranean landscapes (Pareja‐Bonilla et al. 2025). In line with previous studies, we found landscape intensification (i.e., high cover of olive groves in the landscape) to negatively affect wild pollinator abundance (Dainese et al. 2019, Bottero et al. 2023, Laterza et al. 2025). This pattern may reflect the resource scarcity and putative elevated pesticide exposure typical of simplified landscapes, both of which can undermine pollinator populations (Kennedy et al. 2013, Wood et al. 2017, Nicholson et al. 2024).

Ground vegetation cover generally supported ground-dwelling predators. Both spiders and carabids are known to respond to edaphic conditions, being generally sensitive to soil management (Gallé et al. 2019, Manetti et al. 2013, Rowen et al. 2020). The removal of ground cover via mechanical operations can negatively affect these predators both directly, by killing or injuring individuals and interfering with their life cycle, or indirectly, through severe changes in the microhabitat conditions and predator-prey interactions, generally reducing their abundance and diversity, thus potentially impacting their role as ecosystem service providers (Winter et al. 2018, Lee et al. 2019, Angon et al. 2023). Ground cover during summer 2022 was the best predictive period for spider populations, likely because this period follows the main reproductive peak for most Mediterranean species. Summer ground cover management may therefore strongly influence juvenile survival and recruitment, with cascading effects on adult spider populations in the following year (Marc et al. 1999, Angelioudakis et al. 2025). Local management appeared to exert a stronger influence than the surrounding agricultural matrix on spider abundance. This finding partially contrasts with previous research which reported that spider activity density responded to landscape composition (Schmidt and Tscharntke 2005, Nardi et al. 2019). A plausible explanation is that in Mediterranean olive groves, seasonal climatic shifts combined with intensive management disturbances strongly affect ground-dwelling spiders, limiting dispersal and homogenizing assemblages across landscapes, thereby overriding broader landscape effects. Conversely, carabids were supported by the presence of ground cover especially during spring 2023, when most species are present and active (Larsson 1939). This positive effect of ground cover was more evident in complex landscapes (ground cover × landscape composition interaction), whereas carabid populations remained generally more abundant in olive-dominated landscapes, also where low ground vegetation cover was low. This is likely because carabids can readily move across the agricultural matrix and are generally adapted to disturbed habitats, allowing them to thrive even in simplified landscapes (Holland 2002). Overall, preserving ground cover can help to preserve the resident populations of generalist predators also limiting the indirect deterioration of micro-habitats and ecological niches in olive groves (Tamburini et al. 2016a,b, Appenfeller et al. 2022, Müller et al. 2022).

We observed a positive effect of ground cover (early spring 2023) on *P. spumarius* egg predation, especially in landscape richer in seminatural habitat (250 m scale), suggesting that maintaining ground cover within olive groves can enhance the biological control of *P. spumarius* eggs by generalist predators in less intensified landscapes. These results are consistent with previous studies reporting positive effects of ground cover on the egg predation of other olive pests (e.g., *Prays oleae*) (Álvarez et al. 2021). In more complex landscapes, seminatural habitats likely sustain larger and more diverse populations of natural enemies that can spill over into adjacent olive groves, thereby increasing predation pressure when suitable local conditions, such as ground cover, are available (Rusch et al. 2010, Alignier et al. 2014, Dainese et al. 2019). The presence of ground cover during early spring may be particularly important in supporting the generalist predators probably responsible for the predation observed in this study, by providing favorable microclimate conditions, alternative preys, and shelter (Holland et al. 2017). Some egg predation was also observed at low levels of ground cover in more intensified landscape (i.e., lower cover of seminatural habitats). This pattern may partly reflect smaller and less stable natural enemy communities in simplified landscapes, resulting in more variable or opportunistic predation. In addition, the placement of sentinel egg masses along olive groves field edges to avoid damages during soil operations may have masked stronger effects of ground cover management within grove interiors. Interestingly, the ecological drivers of egg predation did not fully matched those of spider and carabid populations, indicating that the observed predation cannot be conclusively attributed to these predator groups. Moreover, ground dwelling predator sampling and egg predation assessment were conducted in different periods (spring and autumn 2023), which further limits our ability to identify the organisms responsible for egg predation later in the season (autumn and/or winter) and highlights the need for targeted studies during autumn and winter. Finally, no parasitoids emerged from the exposed egg masses potentially for two reasons. First, it is possible that these specialized natural enemies may not play a significant role in controlling *P. spumarius* in the studied area (Mesmin et al. 2020). On the other hand, the manipulation of sentinel egg masses may have reduced their attractiveness to parasitoids. Therefore, future studies with longer exposure periods and broader temporal and spatial coverage are needed to fully assess the role of natural enemies in regulating vector populations, also looking to the wild egg masses laid in the field (Mesmin et al. 2020).

In conclusion, our study showed that mandatory removal of ground vegetation cover, implemented to control the spread of *X. fastidiosa* in southern Italy, can generate trade-off between pest control and conservation of beneficial arthropod communities. The removal of ground cover is in fact effective in reducing vector population but may simultaneously have negative impacts on beneficial arthropods (i.e., wild pollinators, spiders, and carabids) and on the biological control of *P. spumarius* eggs. Notably, the importance of herbaceous vegetation conditions varied across seasons, with early spring and spring ground cover emerging as the strongest predictors for most groups, while spider populations responded more strongly to summer conditions. In this context, the timing of ground cover removal appears crucial for mediating its effects on both pest and beneficial arthropod populations in Mediterranean olive groves. Removing ground cover in early spring can effectively suppress vector populations and may have limited impacts on beneficial arthropods, provided that vegetation recovers rapidly afterward. Nevertheless, our study was conducted over a single year, which does not allow to capture potential interannual variability in vector dynamics and associated ecological responses. Longer-term studies would be valuable to determine the consistency of these patterns across years and under different environmental contexts. In addition, our estimates of ground cover did not include a detailed characterization of plant community composition, which can ultimately shape the vector population within the olive groves. Previous research (Antonatos et al. 2021, Bodino et al. 2021), has in fact shown that vegetation composition can influence spittlebug recruitment and activity, and it may also shape communities of beneficial arthropods (e.g., wild pollinators), thereby adding complexity to the trade-offs between pest management and biodiversity conservation. Future research should therefore incorporate both multi-year data and more detailed vegetation assessments to better understand how environmental variability, such as water availability, may mediate these relationships. Finally, we found the characteristics of the agricultural matrix to be pivotal in shaping some beneficial arthropods groups, with complex landscapes supporting their communities and possibly enhancing the predation of the *P. spumarius* eggs within the olive groves, highlighting the importance of a landscape-scale perspective for ecosystem service management.

## Supplementary Material

toag023_Supplementary_Data

## Data Availability

The datasets generated during and/or analyzed during the current study are available from the corresponding author on reasonable request.
