# Supplementary material for: Trade-offs between Xylella fastidiosa vector control and conservation of beneficial arthropods in Mediterranean olive groves
Source: J Econ Entomol. 2026 Feb 25;119(2):728–36. doi: 10.1093/jee/toag023 (PMC13075824; doi:10.1093/jee/toag023)
Supplement: toag023_Supplementary_Data [file toag023_supplementary_data.pdf]

Supplementary information

Trade-offs between Xylella fastidiosa vector control and conservation of beneficial arthropods in Mediterranean olive groves

Ilaria Laterza, Gianvito Ragone, Biagio Tedone, Nicola Bodino, Enrico de Lillo, Daniele Cornara, Giovanni Tamburini

Table S1. Pearson correlations among studied variables.

	Ground cover spring 2022	Ground cover summer 2022	Ground cover autumn 2022	Ground cover early spring 2023	Ground cover spring 2023	Ground cover summer 2023	Ground cover autumn 2023	Olive groves (250 m)	Seminatural habitats (250 m)	Olive groves (500 m)	Seminatural habitats (500 m)	Olive groves (1000 m)
Ground cover summer 2022	0.37 **	–	–	–	–	–	–	–	–	–	–	–
Ground cover autumn 2022	0.31*	0.90***	–	–	–	–	–	–	–	–	–	–
Ground cover early spring 2023	0.06	0.31	0.44*	–	–	–	–	–	–	–	–	–
Ground cover spring 2023	0.02	0.19	0.30	0.67***	–	–	–	–	–	–	–	–
Ground cover summer 2023	0.09	0.42*	0.33	0.20	0.36	–	–	–	–	–	–	–
Ground cover autumn 2023	0.22	0.37	0.42*	0.21	0.36	0.55**	–	–	–	–	–	–
Olive groves (250 m)	-0.09	-0.45 *	-0.31	-0.16	-0.20	-0.52**	-0.25	–	–	–	–	–
Seminatural habitats (250 m)	0.24	0.15	0.08	0.25	0.10	0.23	-0.03	-0.52**	–	–	–	–
Olive groves (500 m)	-0.07	-0.31	-0.14	0.14	0.00	-0.35	-0.07	0.80**	-0.31	–	–	–
Seminatural habitats (500 m)	0.19	-0.01	-0.13	0.14	-0.12	0.10	-0.09	-0.50**	0.85***	-0.36	–	–
Olive groves (1000 m)	-0.05	-0.13	-0.10	-0.48*	-0.20	-0.12	-0.02	-0.18	-0.02	-0.15	0.16	–
Seminatural habitats (1000 m)	0.00	0.02	-0.06	0.22	-0.02	0.16	-0.05	-0.40	0.44	-0.32	0.73***	0.31

Table S2 – Results of model selection for choosing landscape predictor and the appropriate scale for the different models. Besides the best ground vegetation cover predictor previously selected, models were run including the cover of olive groves or seminatural habitats in the landscape at different scales (250 m, 500 m and 1000 m) and compared using the Akaike Information Criterion (AIC). The lowest AIC score for each landscape predictor is in bold.

Landscape predictor	AIC
Xylella fastidiosa vectors	
% olive groves (250 m)	155.80
**% olive groves (500 m)**	**155.02**
% olive groves (1000 m)	156.55
% seminatural habitat (250 m)	156.66
% seminatural habitat (500 m)	155.85
% seminatural habitat (1000 m)	155.71
Wild pollinators	
% olive groves (250 m)	252.44
**% olive groves (500 m)**	**247.25**
% olive groves (1000 m)	252.60
% seminatural habitat (250 m)	254.90
% seminatural habitat (500 m)	254.69
% seminatural habitat (1000 m)	253.19
Spiders	
% olive groves (250 m)	2.95
**% olive groves (500 m)**	**-0.17**
% olive groves (1000 m)	2.46
% seminatural habitat (250 m)	0.33
% seminatural habitat (500 m)	0.67
% seminatural habitat (1000 m)	2.87
Carabids	
**% olive groves (250 m)**	**-19.31**
% olive groves (500 m)	-13.18
% olive groves (1000 m)	-18.05
% seminatural habitat (250 m)	-13.10
% seminatural habitat (500 m)	-14.48
% seminatural habitat (1000 m)	-15.32
Egg predation	
% olive groves (250 m)	-7.95
% olive groves (500 m)	-6.10
% olive groves (1000 m)	-2.25
**% seminatural habitat (250 m)**	**-9.79**
% seminatural habitat (500 m)	-8.91
% seminatural habitat (1000 m)	-8.05

Tab S3 – Details of the models considered in the analyses after the predictor selection based on R^2 and model AIC.

Model	Response variable	Family distribution	Offset	Zero-inflation	Explanatory variables
1	*Xylella fastidiosa* vector	tweedie(link = "log")	-	~1	Ground cover early spring 2023, cover of olive groves (500 m)
2	Wild pollinators	nbinom1	Flower cover		Ground cover spring 2023, cover of olive groves (500 m)
3	Spider	tweedie(link = "log")	-		Ground cover summer 2022, cover of olive groves (500 m)
4	Carabids	tweedie(link = "log")	-		Ground cover spring 2023, cover of olive groves (250 m), ground cover spring 2023 x cover of olive groves (250 m)
5	Egg predation	beta_family()	-		Ground cover early spring 2023, cover of seminatural habitats (250 m), Ground cover early spring 2023 x cover of seminatural habitats (250 m)

FIGURES

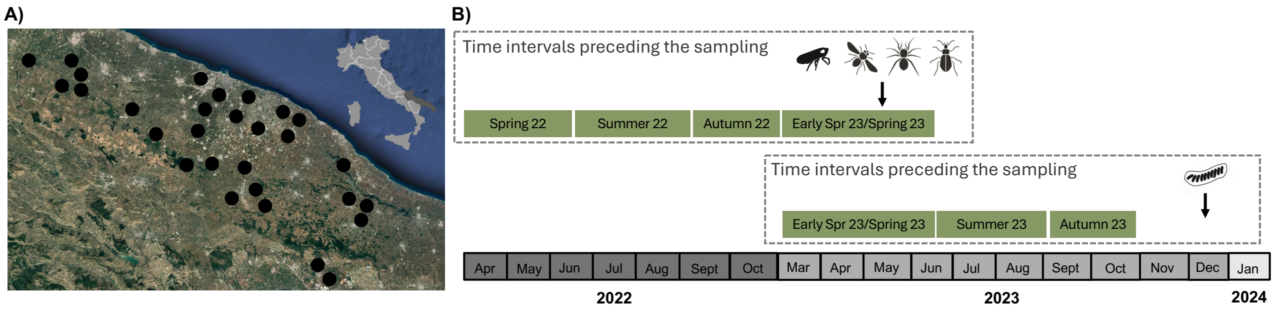

Fig. S1 – Details of the 28 sampling sites selected across the Apulia region (Southern Italy) (A). Time intervals from the year preceding sampling (shown as narrow black lines) considered to assess the effects of ground cover across seasons on the studied organisms (B).

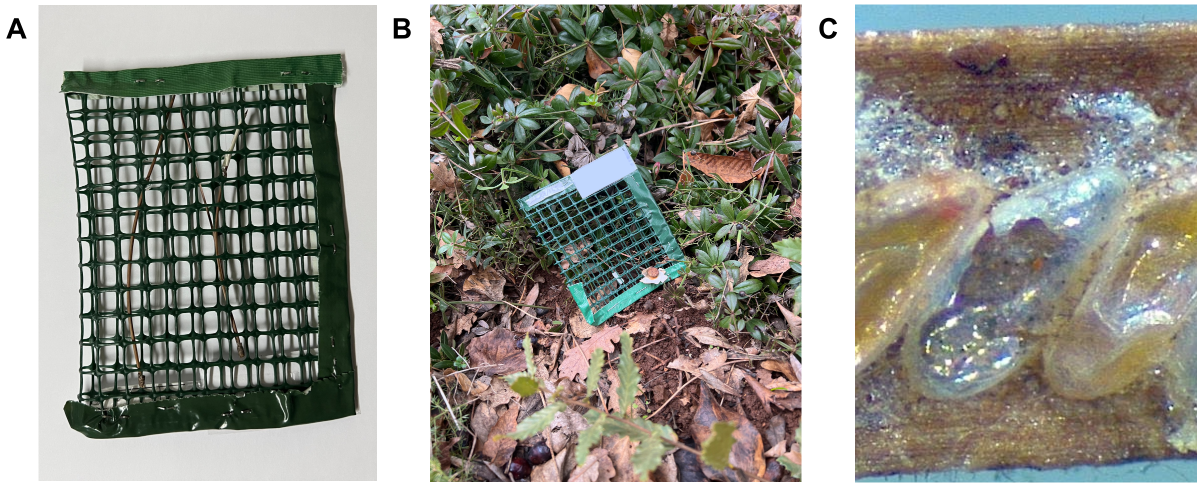

Figure S2– Details of the rigid plastic net used in the egg masses exposure in the field (A, B) and signs of predation found when eggs were retrieved from the field (C).
